# Impulsivity, Social Support and Depression Are Associated With Latent Profiles of Internet Addiction Among Male College Freshmen

**DOI:** 10.3389/fpsyt.2021.642914

**Published:** 2021-03-25

**Authors:** Yue Zhang, Zhuang Liu, Yuhong Zhao

**Affiliations:** ^1^School of Public Health, China Medical University, Shenyang, China; ^2^Department of Clinical Epidemiology, Shengjing Hospital of China Medical University, Shenyang, China; ^3^Clinical Research Center, Shengjing Hospital of China Medical University, Shenyang, China

**Keywords:** freshmen, internet addiction, impulsivity, depression, social support, latent profile analysis

## Abstract

**Background:** The rate of internet addiction is increasing in college students. The first year at college is a particularly vulnerable period for internet addiction. Students' psychological characteristics are likely to play an important role in internet addiction. Our study aimed to assess the relationship between impulsivity, social support, depression and internet addiction among male college freshmen.

**Materials and Methods:** The current study utilized latent profile analysis (LPA) to identify at-risk profiles among 734 college freshmen (100% male) based on their Internet Addiction Test item ratings. We compared the levels of impulsivity, social support and depression among different profiles and investigated whether these variables could predict each latent internet addiction class.

**Results:** LPA resulted in three distinct profiles: the low internet addiction group (42.10%), the moderate internet addiction group (35.70%) and the high internet addiction group (22.20%). Impulsivity and depression increased with internet addiction severity levels, whereas social support was inversely related to the severity of internet addiction. Male freshmen with high impulsivity, low social support and high depression were more likely to be included in the high internet addiction group.

**Conclusion:** This study highlights that impulsivity, social support and depression may predict internet addiction in male college freshmen. Our findings have important practical implications for college educators and counselors in developing interventions for internet addiction.

## Introduction

Along with the intense and rapid development of information technology, the internet has increasingly become a ubiquitous part of daily life. As of June 30, 2020, more than half (62.0%) of the world's population and 59.3% of Chinese people used the internet, which is an increase of 1239 and 3696% since 2000, respectively (1). Recent data from the China Internet Network Information Center (CNNIC) found that 940 million people go online, of which one-fifth are teenagers below 19 years old (2). There is no denying that the internet provides college students with ample learning opportunities.

However, excessive internet usage can introduce serious negative impacts on students' health. Internet overuse may lead to physical problems, including less exercise (3), carpal tunnel syndrome (4), musculoskeletal pain (5), dry eyes, fatigue (6) and poor sleep quality (7), and even self-harm/suicidal behavior (8). Problematic internet use co-occurs with various psychological disorders, such as loneliness (9), lower self-esteem, depression (10) and anxiety (11). Excessive internet use is associated with attention-deficit hyperactivity disorder (ADHD) (12), resulting in poor academic performance (3).

A clinically relevant disorder that may arise from internet overuse is internet addiction, which was proposed by Young in 1996 (13). Moreover, internet addiction is listed as a putative nonsubstance addiction in an appendix of the updated version of the Diagnostic and Statistical Manual of Mental Disorders 5 (DSM-5) (14). A recent meta-analysis comprising 70 studies assessing the prevalence of internet addiction in Chinese college students reported a pooled prevalence of 11.3%, with a wide range of prevalence from 1.9 to 49.41% (15). This reflects the fact that internet addiction has become a serious issue that threatens college students' physical and mental health, and this phenomenon needs greater attention.

Students' psychological characteristics are likely to play an important role in internet addiction and may have lasting harmful effects. Personality traits are one of the important intrinsic factors of addictive behavior, and impulsivity may play a key role in the formation and maintenance of addictive behavior (16, 17). Internet addiction has been associated with impulse control disorder (18), and individuals with internet addiction are more impulsive than those without (19, 20). In addition, in a neuroscience study of 20 males, Park et al. found that 11 internet overusers had altered resting state glucose metabolism in the orbitofrontal cortex and other brain regions, most of which were implicated in impulse control (21). All these results support the hypothesis that the high internet addiction group may have higher impulsivity than those in the low internet addiction group.

Internet addiction is also linked to dissociative symptoms, which are associated with important interpersonal impairments (22, 23). Study also indicates that young people use the internet more frequently than the elderly and need more social inclusion and support from peers (24). Social support is considered to be a predisposing factor that determines a person's core characteristics. It is a basic psychological need that contributes to personal health (25). And social support has been found to be associated with students' mental distress and problematic behaviors (26, 27). A qualitative study of massively multiplayer online games players shows that people often play different roles in the games, which enables young people to obtain social support and meet various emotional needs that are not available in real life (28). These observations lend support to the hypothesis that the lack of social support may increase the susceptibility to internet addiction.

Lack of social support has been reported to cause depression, which may increase the susceptibility to internet addiction (29). Depression has been reported to be one of the most common comorbidities with internet addiction (30). Significant relationships were found between internet addiction and depression (3, 8, 10, 11). A recent study conducted among Japanese University students suggested that depression increased the risk of internet addiction (31). The association between internet addiction and depression can be explained by the emotion enhancement hypothesis, which states that individuals with negative emotions are most likely to seek recreational activities to alleviate stress (32). In this study, we hypothesized that the severity of depression should be highest in the most severe internet addiction group.

Freshmen year is not only an important transition period for young college students to be independent in personality, but also a critical period for development of internet addiction. To gain admission to college, students must study hard in their primary and secondary education, and they have little chance to deal with other things (33). At the beginning of college life, students have ample time and easy access to the internet, which can easily lead to internet addiction (34). Gender differences exist in the development of internet addiction (35). Previous research has shown that male college students have a greater possibility of developing internet addiction than females (36–40). Male students exhibit highly addictive patterns toward internet gaming and are more likely to indulge in the internet when they lack parental supervision (41, 42). Compared with internet addiction in female group, internet addiction in male group has greater association with school burnout (43). Exploring the risk factors of internet addiction in male college freshmen may assist in preventing their internet addiction and enabling the provision of interventions. To date, only a handful of studies have examined internet addiction among male freshmen (44). Therefore, our study sought to examine the relationship between impulsivity, social support, depression and internet addiction to gain a deeper understanding of the effects of internet addiction on male freshmen.

Students in most existing studies have been divided into different categories according to their total score for internet addiction disorder. However, the cut-off value of each subgroup has not been uniform in these studies, and this value-oriented research method does not fully take into account individual differences. Therefore, a person-oriented approach may be a valuable way to examine the response patterns of participants and establish different potential classes based on the average scores of observed indicator variables (45). Latent profile analysis (LPA) is a person-oriented classification method that models continuous indicators. LPA allows for the estimation of profile-specific means, variances, and covariances and facilitates a more granular examination of heterogeneity within and between potential levels (45). LPA can be used as a means to identify high-risk individuals to implement tailored interventions (46).

The main purpose of the present study was to explore distinct classes of male freshmen based on the internet addiction test using mixture modeling (LPA). We evaluated the differences in demographic and psychological characteristics between different groups of male freshmen. Furthermore, we explored whether impulsivity, social support and depression predicted the likelihood of class membership. Three main hypotheses guided our study. Hypothesis 1: The high internet addiction group may have higher impulsivity than those in the low internet addiction group. Hypothesis 2: The lack of social support may increase the susceptibility to internet addiction. Hypothesis 3: The severity of depression should be highest in the most severe internet addiction group. Our results may be useful for health care professionals to design appropriate health services and effective strategies to reduce internet addiction among college students.

## Materials and Methods

### Participants and Procedure

A cross-sectional survey was conducted at a mid-sized public college in Liaoning during the autumn semester of 2019. Inclusion criteria included: the first-year students on campus, male students and willing to participate in the study. Exclusion criteria included: the presence of chronic diseases and psychiatric disorders. Before participation, the purpose of this study was introduced to the students. Confidentiality and anonymity were maintained throughout the entire study, and students could withdraw from the study at any time. Informed consent was attained from all students prior to participation. Students were allocated 20–30 min to complete the self-reported questionnaires and were rewarded with gifts.

The final sample of our study comprised 734 college freshmen from several different departments. The students ranged from 16 to 23 years old, with an average age of 19.74 years (*SD* = 1.38). Over half (64.30%) of the male students were from an only-child family, and the majority of participants resided with both biological parents (92.20%). A total of 87.30% of the students had a paternal education level less than senior high school, and 67.80% had a maternal education level less than senior high school. The majority of the respondents were rural residents (56.00%), 26.20% lived in urban areas, and 17.80% came from towns. Only 9.00% of the students had good household economic conditions, 55.80% had medium-level economic conditions, and the rest had low household economic conditions.

### Measurements

#### Demographics

Participants were asked to complete a questionnaire on their sociodemographic characteristics, such as age and family background. The students' family background was evaluated based on the following four aspects: whether the student was an only child, paternal and maternal educational levels, family residence and household economic conditions.

#### Internet Addiction Test (IAT)

The IAT designed by Young KS (47) has been widely used to screen whether users are addicted to the internet as well as the severity of their addiction. The IAT consists of 20 items that are rated on 5-point scales ranging from 1 (very rarely) to 5 (very frequently). The total score ranges from 20 to 100, with higher total scores indicating more severe internet addiction (48). The internal consistency for the IAT was good in the current sample (alpha = 0.928).

#### Barratt Impulsiveness Scale 11 (BIS-11)

The BIS-11 was utilized to assess participants' impulsive traits by rating their frequency of impulsive behaviors or cognitions on a scale from 1 (rarely/never) to 4 (almost always) (49). The BIS-11 is composed of 30 items and is divided into three subscales: motor impulsiveness (MI), cognitive impulsiveness (CI) and nonplanning impulsiveness (NPI) (20). Higher scores are indicative of higher levels of impulsiveness. The BIS-11 has previously been validated in China (50). In the current study, the Cronbach's alpha coefficient for the BIS-11 was 0.831.

#### Social Support Rating Scale (SSRS)

We adopted the SSRS designed by Xiao SY to measure the perceived adequacy of support *via* self-report, including objective support (OS), subjective support (SS), and social support availability (SUA) (51). In our study, “Colleague, relative and leader” in the questionnaire was changed to “Classmate, relative and teacher” to make the scale suitable for measuring students. The scores of the three dimensions were added to generate a total score of 11 to 62. The higher the total score, the greater the respondent's likelihood of obtaining social support. The SSRS showed moderate internal consistency with a Cronbach's alpha coefficient of 0.743.

#### The Center for Epidemiologic Studies Depression Scale (CESD)

The CESD was administered in the present study to evaluate the depressive status of the students. The CESD includes 20 items that indicate how frequently common depressive symptoms occurred in the previous week (52). It assesses depression across 4 domains: depressed affect (DA), positive affect (PA), somatic and slow activity (SRA) and interpersonal (IP) (52). Responses to each question were provided on a 4-point Likert scale (ranging from “0 = never” to “3 = almost all the time”). Four items (e.g., “During the past week I enjoyed life”) were reverse coded, which were accounted for in the total score. The overall scores range from 0 to 60, with higher scores indicating a greater degree of depression symptoms. The depression measure had good reliability in our study (alpha = 0.876).

#### Statistical Analysis

Correlation analyses of our primary variables were performed prior to conducting LPA. We used Mplus Version 7.0 for LPA based on the IAT items to evaluate distinct profiles of internet addiction. In our study, LPAs with one to eight class solutions were considered sequentially. Identifying the model with the optimal number of latent classes is a critical step in the analysis. To select the best-fitting LPA model, we examined the following statistical indices: Akaike's Information Criterion (AIC) (53), Bayesian Information Criterion (BIC) (54), adjusted BIC (aBIC) (55), bootstrap likelihood ratio test (BLRT) (56), adjust Lo-Mendell-Rubin likelihood ratio test (aLMR) and entropy (57). The AIC, BIC and aBIC are descriptive statistics of the fitness of the LPA model; lower values indicate better fit. Significant differences in the BLRT and aLMR between class models (e.g., *n* vs. *n*-1) are indicative that the *n* class model is a better fit than the *n*-1 one, and a better model is indicated with the last significant BLRT and aLMR (57). Entropy, ranging from 0 to 1, is used to assess the accuracy of classification, with a score of 0.80 or higher indicative of adequate classification precision (58).

After determining the best-fitting model with the optimal number of classes, cross-class comparisons were performed according to the demographic and psychological factors with SPSS Statistics (version 22). The comparisons were carried out with nonparametric statistics using χ^2^ tests and ANOVAs for categorical and continuous variables, respectively. *Post hoc* analyses with Bonferroni tests were conducted when significant differences were found. The psychological characteristics (impulsivity, social support and depression scores) that may be associated with category membership were assessed using the three-step maximum likelihood method, which analyzed the predictors of potential categories and took into account the classification errors in the original measurement model. A significance level of alpha ≤ 0.05 was considered significant.

## Results

### Descriptive Statistics

The internet addiction severity averaged 39.530 (*SD* = 13.215). The BIS-11 mean score was 65.310 ± 11.110. The average score on the SSRS was 39.580 ± 7.078, and the average score on the CESD was 16.980 ± 8.844. The relationships between impulsivity, social support, depression and internet addiction were tested with bivariate correlation analyses along with age. As described in Table 1, IAT scores were positively correlated with BIS motor impulsiveness, nonplanning impulsiveness, CESD depressed affect, positive affect, somatic and slow activity and interpersonal impulsiveness. Moreover, IAT scores were negatively correlated with SSRS objective support, subjective support, and social support availability.

**Table 1:** Bivariate correlation analysis of study variables in the males.

**Variables**	**1**	**2**	**3**	**4**	**5**	**6**	**7**	**8**	**9**	**10**	**11**	**12**	**13**	**14**	**15**
1.age	1.000														
2.IAT	0.000	1.000													
3.BIS-11	−0.022	0.506^**^	1.000												
4.MI	0.012	0.467^**^	0.876^**^	1.000											
5.CI	−0.068	0.372	0.815^**^	0.571^**^	1.000										
6.NPI	−0.013	0.473^**^	0.920^**^	0.710^**^	0.639^**^	1.000									
7.SSRS	0.027	−0.233^**^	−0.282^**^	−0.264^**^	−0.212^**^	−0.258^**^	1.000								
8.OS	−0.036	−0.179^**^	−0.243^**^	−0.227^**^	−0.182^**^	−0.222^**^	0.761^**^	1.000							
9. SS	0.029	−0.174^**^	−0.205^**^	−0.187^**^	−0.162^**^	−0.187^**^	0.870^**^	0.441^**^	1.000						
10. SUA	0.089^*^	−0.202^**^	−0.218^**^	−0.214^**^	−0.150^**^	−0.202^**^	0.618^**^	0.319^**^	0.352^**^	1.000					
11.CESD	0.059	0.591^**^	0.521^**^	0.528^**^	0.378^**^	0.453^**^	−0.331^**^	−0.261^**^	−0.244^**^	−0.282^**^	1.000				
12. DA	0.045	0.569^**^	0.442^**^	0.467^**^	0.325^**^	0.364^*^	−0.267^**^	−0.179^**^	−0.212^**^	−0.245^**^	0.902^**^	1.000			
13. PA	0.063	0.159^**^	0.302^**^	0.289^**^	0.186^**^	0.300^**^	−0.287^**^	−0.282^**^	−0.205^**^	−0.176^**^	0.473^**^	0.163^**^	1.000		
14. SRA	0.023	0.564^**^	0.461^**^	0.455^**^	0.350^**^	0.400^**^	−0.216^**^	−0.163^**^	−0.140^**^	−0.235^**^	0.872^**^	0.773^**^	0.167^**^	1.000	
15. IP	0.064	0.446^**^	0.348^**^	0.354^**^	0.268^**^	0.291^**^	−0.250^**^	−0.204^**^	−0.196^**^	−0.180^**^	0.743^**^	0.642^**^	0.201^**^	0.622^**^	1.000

* *p < 0.05*;

** *p < 0.01*.

### LPA Results

The model fit statistics for the latent profile models ranging from 1 to 8 classes are given in Table 2. As shown, AIC, BIC and aBIC leveled off slightly after the 3-class model was assigned, indicating little improvement. Given that the BLRT *p*-value was significant across the 7 class models, we chose the 3-class model as the best-fitting model based on the aLMR *P*-value. Moreover, the aLMR *P*-value was significant for the 3-class model and nonsignificant for the 4-class model. Specifically, both AIC and BIC were smaller for class three than for class two. In addition, the entropy value for the 3-class model (0.900) suggested a high level of precision in assigning students to their appropriate groups. Therefore, the 3-class model was determined to be the best-fitting parsimonious model.

**Table 2:** Model fit statistics for latent profile analysis in the males (*N* = 734).

**Classes (*n*)**	**AIC**	**BIC**	**aBIC**	**BLRT *P-*value**	**aLMR *P*-value**	**Entropy**
1	42308.924	42492.864	42365.851	–	–	–
2	37797.415	38077.924	37884.229	<0.001	<0.001	0.950
**3**	**36736.667**	**37113.744**	**36853.367**	** <0.001**	** <0.001**	**0.900**
4	36363.780	36837.427	36510.367	<0.001	0.392	0.885
5	36090.236	36660.452	36266.710	<0.001	0.678	0.915
6	35865.015	36531.799	36071.375	<0.001	0.251	0.890
7	35750.624	36513.976	35986.871	<0.001	0.472	0.902
8	35699.030	36558.952	35965.164	1.000	0.812	0.897

*AIC, Akaike's Information Criterion; BIC, Bayesian Information Criterion; aBIC, adjusted BIC; BLRT, bootstrap likelihood ratio test; aLMR, adjust Lo-Mendell-Rubin likelihood ratio test; –not applicable. The bold values means the best-fitting parsimonious model*.

Class 1, “low internet addiction group,” had the biggest class size (*N* = 309; 42.10%), which exhibited low risks of internet addiction. Class 2 (*N* = 262; 35.70%) was labeled “moderate internet addiction group”, which members having moderate potential of internet addiction. Class 3 (*N* = 163; 22.20%) represented individuals with higher scores of IAT, this suggested that Class 3 students tended to seriously indulge in internet use. Hence, this class was named “high internet addiction group.”

### Demographic Results

Given that the 3-class model provided meaningful internet addiction classifications for male freshmen students, we assessed differences among the three groups based on sociodemographic characteristics. There were no statistically significant differences among the three groups in the students' family background factors, including whether the student was an only child (χ^2^ = 0.923, *P* = 0.630), paternal and maternal educational levels (χ^2^ = 4.708, *P* = 0.095 for paternal; χ^2^ = 2.969, *P* = 0.227 for maternal), family residence (χ^2^ = 3.269, *P* = 0.514) and household economic conditions (χ^2^ = 0.341, *P* = 0.843).

### Cross-Class Comparisons

To examine the differences in impulsivity, social support and depression across the three groups, ANOVAs were conducted on the total scores of IAT, BIS-11, SSRS, CESD and their subfactors; the results are presented in Table 3. Significant differences in the total scores of the four scales and the scores on the subfactors emerged across three groups in the participants. These findings demonstrate that the classes were correctly clustered according to the specific features of internet addiction. *Post hoc* interclass comparisons revealed that the high internet addiction group was more likely to score higher on internet addiction, impulsivity and depression and lower on social support.

**Table 3:** Mean comparisons across groups on IAT, BIS-11, SSRS and CESD.

**Variables**	**Low (*N* = 309)**	**Moderate (*N* = 262)**	**High (*N* = 163)**	***P*-value**	***F***	***Post hoc***
IAT	27.540 ± 4.800	41.750 ± 4.702	58.650 ± 7.777	<0.001	1695.624	H>M>L
BIS-11	59.580 ± 11.366	66.730 ± 8.796	73.880 ± 6.987	<0.001	121.334	H>M>L
MI	19.050 ± 4.235	21.370 ± 3.299	23.970 ± 3.352	<0.001	94.334	H>M>L
CI	21.650 ± 3.597	23.440 ± 2.882	24.820 ± 2.893	<0.001	55.923	H>M>L
NPI	18.880 ± 5.065	21.930 ± 4.168	25.090 ± 3.535	<0.001	107.083	H>M>L
SSRS	40.990 ± 7.595	39.530 ± 5.716	36.980 ± 7.335	<0.001	17.926	L>M>H
OS	9.360 ± 3.060	9.010 ± 2.388	8.100 ± 3.232	<0.001	10.362	L≈M>H
SS	23.370 ± 4.199	22.880 ± 3.720	21.460 ± 4.546	<0.001	11.618	L≈M>H
SUA	8.260 ± 2.106	7.630 ± 1.745	7.420 ± 1.940	<0.001	12.360	L>M≈H
CESD	12.770 ± 7.569	17.020 ± 6.475	24.940 ± 8.960	<0.001	138.428	H>M>L
DA	4.330 ± 3.585	6.270 ± 3.108	9.620 ± 4.798	<0.001	106.548	H>M>L
PA	3.900 ± 2.908	4.220 ± 2.277	5.330 ± 2.663	<0.001	15.991	H>M≈L
SRA	3.670 ± 2.702	5.280 ± 2.414	7.620 ± 3.333	<0.001	109.799	H>M>L
IP	0.870 ± 1.151	1.250 ± 1.091	2.370 ± 1.536	<0.001	80.181	H>M>L

*L, low internet addiction group; M, moderate internet addiction group; H, high internet addiction group*.

The low internet addiction group exhibited the lowest level of internet addiction and the lowest IAT scores. The moderate internet addiction group exhibited IAT scores that were significantly higher than those of the low internet addiction group and significantly lower than those of the high internet addiction group (*P* <0.001). Thus, the level of internet addiction in descending order was the high internet addiction group, the moderate internet addiction group, and the low internet addiction group. An overall *F*-test revealed that impulsivity was significantly different across the three groups, including motor impulsiveness, cognitive impulsiveness and nonplanning impulsiveness (all *P* <0.001). The high internet addiction group had the highest scores on the total scores of BIS-11 and its three subscales, followed by the moderate internet addiction group. The low internet addiction group exhibited the lowest scores. In contrast, the more severe internet addiction group demonstrated lower levels of social support. The low internet addiction group reported the most social support, followed by the moderate internet addiction group, while the high internet addiction group received the least social support. Similar results were observed in the SSRS subscales. However, significant differences between the low internet addiction group and the moderate internet addiction group were not found in objective support and subjective support. The moderate internet addiction group and the high internet addiction group did not differ significantly in social support availability. With the exception that the difference between the moderate and high internet addiction groups was not statistically significant, the general pattern of depression was similar to that of impulsivity.

### Covariate Results

Table 4 presents the effects of impulsivity, social support and depression on group membership using the low internet addiction group as the reference group. Compared to the low internet addiction group, the moderate and high internet addiction groups had significantly higher scores for both impulsivity and depression. For each point of increase in impulsivity, the odds ratios of being in the moderate internet addiction group increased from 1,000 to 1,071 and the odds ratios of being in the high internet addiction group increased to 1,186. Each added point for depression was associated with a 9.7% increased likelihood of being in the moderate internet addiction group compared with the low internet addiction group and a 24.9% increased likelihood of being in the high internet addiction group compared with the low group. Male freshmen with higher values for social support were less likely to be in the moderate and high internet addiction groups than the low internet addiction group. Based on odds ratios, each added point for social support was associated with a 3.5% decreased likelihood of being in the moderate internet addiction group compared with the low group and a 7.6% decreased likelihood of being in the high internet addiction group compared with the low group.

**Table 4:** Predictors of group membership with low internet addiction group as the reference group.

**Covariate**	**Moderate internet addiction group**				**High internet addiction group**			
	***B***	***SE***	***OR***	***P***	***B***	***SE***	***OR***	***P***
Impulsivity	0.069	0.013	1.071	<0.001	0.171	0.018	1.186	<0.001
Social support	−0.036	0.014	0.965	0.010	−0.079	0.018	0.924	<0.001
Depression	0.093	0.015	1.097	<0.001	0.222	0.021	1.249	<0.001

## Discussion

In present study, we concluded that the 3-class model was the most effective and accurate in terms of presenting different degrees of internet addiction in male freshmen. There was a clear order of three underlying internet addiction groups, low, moderate and high internet addiction. Male freshmen in the low internet addiction group had lower scores on all items of the IAT scale, and showed a lower risk of internet addiction. They had the lowest scores for impulsivity and depression and the highest scores for social support. The situation in the high internet addiction was completely opposite. Impulsivity, social support and depression may predict internet addiction in male college freshmen. Our study provided a reference for internet addiction disorder interventions among male freshmen.

We empirically found that male college freshmen could be divided into 3 classes indicating their internet addiction. Most of the male freshmen (42.10%) were low in internet addiction, and only a few of them (22.20%) were more severe. Our results support the studies conducted in Korean (42) and American (59) college students, which also found three groups, despite the presence of smartphone addiction in their studies. However, the results of the present study are inconsistent with some previous studies, which were conducted in Germany and Korea and found five (60) and six (61, 62) classes, respectively. Our results may differ because we used eastern samples and used item responses rather than scale scores.

Our data indicated that impulsivity was positively correlated with internet addiction, and the scores for impulsivity increased as the severity of internet addiction in male college freshmen students increased. This research verifies Hypothesis 1 and further supports the assumption that impulsivity is regarded as a marker of susceptibility to internet addiction, consistent with numerous previous studies (17, 63–67). These findings suggest that individuals with internet addiction display an elevated tendency toward most impulsivity traits compared to non-Internet addiction. However, a case-control study presented no significant association between impulsivity and internet addiction in Chinese male freshmen (44), inconsistent with the aforementioned findings. The divergence in these data might be partly due to different methodologies, such as the former used of the Chinese College Student Mental Health Scale (CCSMHS) and other studies used BIS.

Interestingly, in our study, the correlation analysis showed that motor impulsiveness and nonplanning impulsiveness were positively correlated with internet addiction, whereas cognitive impulsiveness was not correlated with internet addiction. This finding suggests that specific traits of impulsivity might serve as important factors in identifying male freshmen at increased risk for internet addiction. The BIS-11 captures the multidimensional personality traits of impulsivity: motor impulsiveness, the tendency to act recklessly and to be easily attracted by the outside world; cognitive impulsiveness, the tendency to be unable to concentrate on things; and nonplanning impulsiveness, the tendency not to consider and plan for the future (68, 69). Compared to low impulsivity males, high impulsivity males may often be short-sighted and only consider current interests when making decisions. When using the internet, they may only think of the immediate pleasure and ignore the adverse consequences of long-term use of the internet, which leads to excessive internet use and eventually to internet addiction. And the drive to succeed in online games and activities may also drive ambitious male students to be more prone to internet addiction (70). Moreover, numerous neuroimaging studies have indicated that compared with normal individuals, high-impulsive individuals have certain reduced activities in the lateral and medial prefrontal lobe (71) and right frontal lobe (72). These brain regions play critical roles in cognitive control (73), decision-making (74) and emotional regulation (75). Therefore, high-impulsive male freshmen may have more difficulty controlling their online behaviors, which in turn leads to internet addiction.

Our results showed a significant inverse correlation between internet addiction and social support (including objective support, subjective support, and social support availability), which validated Hypothesis 2. With each additional point of social support, the probability of male freshmen being assigned to the moderate-risk group and the high-risk group decreased by 3.5 and 7.6%, respectively. The results are in conformity with the extant literature that studied the relationship between social support and internet addiction (76–78). Study showed that participants with higher scores in receiving social support reported the lowest tendencies toward internet use disorder (76). Obviously, social support is a potential factor for internet addiction (77, 78). An important characteristic of Chinese college students is that most of them are living independently from their parents for the first time, and their main social environment is composed of peers. In such an environment, many male freshmen cannot deal with interpersonal relationships well, and those with poor interpersonal relationships often lack social support, which leads them to turn to the internet to make up for the lack of social support (79). The internet may provide students with a social support platform to help them relieve psychological pressure (80). Consequently, students are increasingly dependent on the internet, ultimately resulting in internet addiction behavior. This phenomenon is in line with the findings, which found that online social support was positively associated with internet addiction, whereas offline social support ties were negatively associated with internet addiction (81). In addition, social support is a robust coping resource that may provide emotional assistance to cope with stress and thus directly or indirectly reduce students' vulnerability to internet addiction (82).

In terms of the relationship between depression and internet addiction, we found that depression may worsen internet addiction behavior in male freshmen, supporting Hypothesis 3. After covariate adjustment, the depression scores of the moderate internet addiction group and the high internet addiction group were higher than those of the low internet addiction group. This finding corroborates some studies on internet addiction in other regions, such as Japan (31), Taiwan (83) and Poland (84), which have reported that people with depression are susceptible to internet addiction. Males who suffer from depression may have low self-esteem and feel a strong need to be accepted (85). Through the internet, information could be transmitted quickly, making it possible for people around the world to interact with each other. The Internet could provide a virtual space where they may express themselves, release pressure and regain confidence (86). Therefore, male freshmen may cope with negative mood through excessive internet use. This phenomenon may somewhat explain why depressed students can easily develop internet addiction. Moreover, pathological use of the internet is detrimental to mental health and can exacerbate depression (87). Therefore, depression and internet addiction may contribute to each other. This is demonstrated by a study examining the bidirectional predictions between depression and internet addiction (88). In treatment and diagnosis, it is necessary to remember that they are mutually aggravating.

## Limitations and Implications

Some strengths and limitations of this study should be noted. First, because this study was a cross-sectional design, it was not possible to make conclusive statements about the temporal order between psychological characteristics and internet addiction. A longitudinal study should be designed in further explorations. However, appropriate analysis of the cross-sectional data represents a useful initial step in identifying associations between internet addiction and impulsivity, social support and depression. Second, information on the exposure variable was collected *via* self-reporting and is subject to recall or report bias. Third, because the participants were limited to male college freshmen, the results may not apply to other samples. However, the results of study suggest that male freshmen with features of impulsivity, less social support and depression could be a valuable target for intervention to reduce internet addiction. Our results can help college freshmen form positive internet use behaviors at a critical stage.

## Conclusion

Our study offers insights into subgroups of symptom presentations from IAT ratings and deepens the understanding of the psychological characteristics of male freshmen in the field of internet addiction. The severity of internet addiction is affected by male impulsivity, social support and depression. It is important for counselors, educators and parents to design interventions. Colleges can design comprehensive intervention measures according to the characteristics of internet addiction in different subgroups, including psychological intervention and family intervention. On the one hand, this can help students understand and alleviate their own negative emotions; on the other hand, it can teach students appropriate social skills and enable them to obtain social resources to reduce the possibility of internet addiction.

## Data Availability Statement

The raw data supporting the conclusions of this article will be made available by the authors, without undue reservation.

## Ethics Statement

This study was conducted in accordance with the Declaration of Helsinki, and the protocol was approved by the Ethics Committee of China Medical University. All participants were informed about the study and all provided informed consent.

## Author Contributions

YHZ designed the study. YZ and ZL conducted the survey. ZL conducted the statistical analysis. YZ wrote the first draft of manuscript. All authors revised the initial manuscript and finalized this paper for submission and read and approved the final manuscript.

## Conflict of Interest

The authors declare that the research was conducted in the absence of any commercial or financial relationships that could be construed as a potential conflict of interest.
